# Narratives of community engagement: a systematic review-derived conceptual framework for public health interventions

**DOI:** 10.1186/s12889-017-4958-4

**Published:** 2017-12-11

**Authors:** Ginny Brunton, James Thomas, Alison O’Mara-Eves, Farah Jamal, Sandy Oliver, Josephine Kavanagh

**Affiliations:** 1 Department of Social Science, Evidence for Policy and Practice Information and Coordinating (EPPI)-Centre, Social Science Research Unit, UCL Institute of Education, University College London UK, 18 Woburn Square, London, WC1H 0NR UK; 2 National Institute for Health and Care Excellence, Manchester, UK

**Keywords:** Community engagement, Public health, Health promotion, Systematic review, Conceptual framework

## Abstract

**Background:**

Government policy increasingly supports engaging communities to promote health. It is critical to consider whether such strategies are effective, for whom, and under what circumstances. However, ‘community engagement’ is defined in diverse ways and employed for different reasons. Considering the theory and context we developed a conceptual framework which informs understanding about what makes an effective (or ineffective) community engagement intervention.

**Methods:**

We conducted a systematic review of community engagement in public health interventions using: stakeholder involvement; searching, screening, appraisal and coding of research literature; and iterative thematic syntheses and meta-analysis. A conceptual framework of community engagement was refined, following interactions between the framework and each review stage.

**Results:**

From 335 included reports, three products emerged: (1) two strong theoretical ‘meta-narratives’: one, concerning the theory and practice of empowerment/engagement as an independent objective; and a more utilitarian perspective optimally configuring health services to achieve defined outcomes. These informed (2) models that were operationalized in subsequent meta-analysis. Both refined (3) the final conceptual framework. This identified multiple dimensions by which community engagement interventions may differ. Diverse combinations of intervention purpose, theory and implementation were noted, including: ways of defining communities and health needs; initial motivations for community engagement; types of participation; conditions and actions necessary for engagement; and potential issues influencing impact. Some dimensions consistently co-occurred, leading to three overarching models of effective engagement which either: utilised peer-led delivery; employed varying degrees of collaboration between communities and health services; or built on empowerment philosophies.

**Conclusions:**

Our conceptual framework and models are useful tools for considering appropriate and effective approaches to community engagement. These should be tested and adapted to facilitate intervention design and evaluation. Using this framework may disentangle the relative effectiveness of different models of community engagement, promoting effective, sustainable and appropriate initiatives.

## Background

Community engagement has been advanced as a useful strategy for improving people’s health and as a means of enabling people who lack power to gain control over their lives – and thereby improve their own health. In many countries, it is part of clinical guidance [1] and the national strategy for promoting public health [2], and is a prominent feature in the policies and mission statements of local healthcare services. Whilst high on the public health care agenda, there is inconsistency in the terms used to describe it, the meanings ascribed to it, and the rationales underpinning the stated ‘need’ for it. Related to this, the conceptual and moral breadth of community engagement poses challenges to those planning and commissioning health services: should they use community engagement in a given situation? If so, how should they do this? And how can they know which approach would be most suitable? In order to begin framing answers to some of these questions, we need to understand what community engagement is, where the concept came from, and how it is proposed to work. This will reveal how some of the different perspectives and agendas that have coalesced around the term “community engagement”; and how different approaches to engagement are thought to impact on people’s health.

To understand these issues, we conducted a systematic review of the literature around community engagement. The systematic review design is well-suited to the research questions. As well as addressing intervention effectiveness, systematic reviews present an opportunity to take stock and examine some of the assumptions underlying research activity. They can ‘recast’ the literature, by analysing how research is located within particular conceptual and ethical frameworks, and tracing the development of thought over time [3, 4].

This paper presents the findings from a synthesis that examined the theory underpinning, factors involved in, models of change, and evidence for, community engagement in terms of its impacts on a wide range of health outcomes. This was one component of a larger multi-method systematic review project, which contained four different syntheses of community engagement in addition to the theoretical synthesis presented here: a map of theoretical and effectiveness community engagement literature, a thematic synthesis of processes, a meta-analysis of trials, and an economic analysis of costs and resources. The complete project findings are reported elsewhere [5]. In this paper, we report on the research synthesis which examined the theoretical and empirical literature to identify the key characteristics of community engagement interventions, organising them into a new conceptual framework which encapsulates the wide range of understandings and perspectives around community engagement, and how these are implemented in practice. Within this overarching conceptual framework, specific models were identified, enabling us to distinguish how different approaches might impact on people’s health.

## Methods

The conceptual framework described here is part of a multi-method systematic review which aimed to identify: community engagement approaches that improve the health of disadvantaged populations or reduce inequalities in health; the populations and circumstances in which they ‘work’; and associated costs. Review stages included: stakeholder involvement; literature searching, screening studies for eligibility, critical appraisal and coding of studies; and synthesis. Each stage is described briefly below, with further detail available in the full report [5].

### Aims and research questions

The aim of this paper is to describe the development of a conceptual framework and models arising from an iterative synthesis of both papers discussing community engagement theory and informed by the broader review. The research questions for theory synthesis were:

1. What is the range of models and approaches underpinning community engagement?
2. What are the mechanisms and contexts through which communities are engaged?

We define a conceptual framework or theory here to be a working hypothesis of key concepts, constructs and their potential interactions [6]. Models, mechanisms or theories of change are considered to be synonymous; these focus in on single specific hypothesised processes drawn from that wider conceptual framework to identify how one phenomenon influences another [7].

### Stakeholder involvement

Community engagement researchers, policy-makers and other professionals were invited to take part in our Advisory group. They informed the conceptual framework by providing key research articles on community engagement, commenting on iterations of our developing conceptual framework, and advising on potential synthesis approaches.

### Searching

To locate all possible research on community engagement initiatives, systematic reviews and primary studies evaluating community engagement interventions reporting health outcomes were sought from specially-selected registers of research, including: the Cochrane and Campbell Libraries; the National Institute for Health Research (NIHR) Health Technology Assessment (HTA) programme website and HTA database; and the Database of Promoting Health Effectiveness Reviews (DoPHER). The majority of these specialist registers were populated using rigorous systematic review search methods. In addition, theoretical and “position pieces” on community engagement were sought using more iterative processes (including following citation trails and website searching). We adopted an innovative search strategy to locate this literature, utilising the structured data often presented in systematic review reports, as reviews inconsistently described employing a community engagement strategy in their title and abstract alone [8].

### Screening for eligibility

To inform the theory synthesis, we identified first any theoretical literature from within our set of retrieved studies, adopting a ‘purposive’ search and inclusion strategy appropriate to gathering concepts, rather than the more traditional approach of exhaustively accumulating all literature on the topic [9]. ‘Theoretical literature’ was considered any research paper discussing theoretical issues around community engagement. Thus, potentially useful theoretical papers were ‘included’ regardless of whether they met other aspects of the inclusion criteria (e.g., they did not necessarily have to report relevant outcomes).

We next screened for intervention studies. To be eligible for inclusion in the broader review, studies had to meet the following criteria:

- published after 1990;
- a systematic review or primary research study;
- an outcome or process evaluation;
- an intervention relevant to community engagement;
- written in English;
- measure and report health or community outcomes;
- characterise study populations or report differential impacts in terms related to social determinants of health; and
- contain health or health-related outcomes, and/or process data.

### Study appraisal and selection

Papers were included if they contributed to our understanding of community engagement’s theoretical foundation(s). This is in line with “purposive” sampling strategies often used in qualitative research. Here, the “logic and power of purposeful sampling lie[s] in selecting information-rich cases for study in depth” [emphasis in original, p.230 [10]]. For example, in the course of the review, we found many studies which examined the recruitment of ‘peers’ to deliver the intervention. We did not need to ‘include’ every study on peer delivery to inform the theory synthesis however, since once their key characteristics had been identified in the first few papers examined, additional examples of the same intervention strategy did not contribute any new concepts. Using this approach, team members identified a subset of theoretically-focused papers containing examplars for every community engagement strategy.

### Coding and synthesis

#### Conceptual framework development and examination of theory

**Fig. 1:**
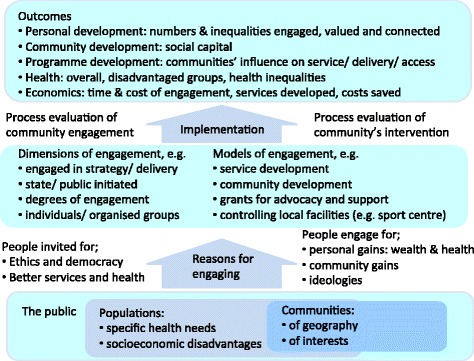
Initial conceptual framework

Using a diverse literature to develop an overarching conceptual framework involved three main tasks: the identification of key concepts and theoretical stances; consideration of how they relate to one another – both within and between studies; and the development of an explanatory theory(the final framework), within which different models were located. This is an iterative process where initial conceptual frameworks were drawn up, ‘tested’ against existing and new literature, and revised. Using methods derived from framework synthesis [11, 12], we began with one framework (see Fig. 1), which had informed our initial research proposal and protocol.

This was changed significantly during the review. As new theoretical and evaluation papers were assessed, the framework was examined to see: whether it could adequately encompass the new paper; if new detail was needed, or if a fundamental reappraisal of its structure was necessary.

**Fig. 2:**
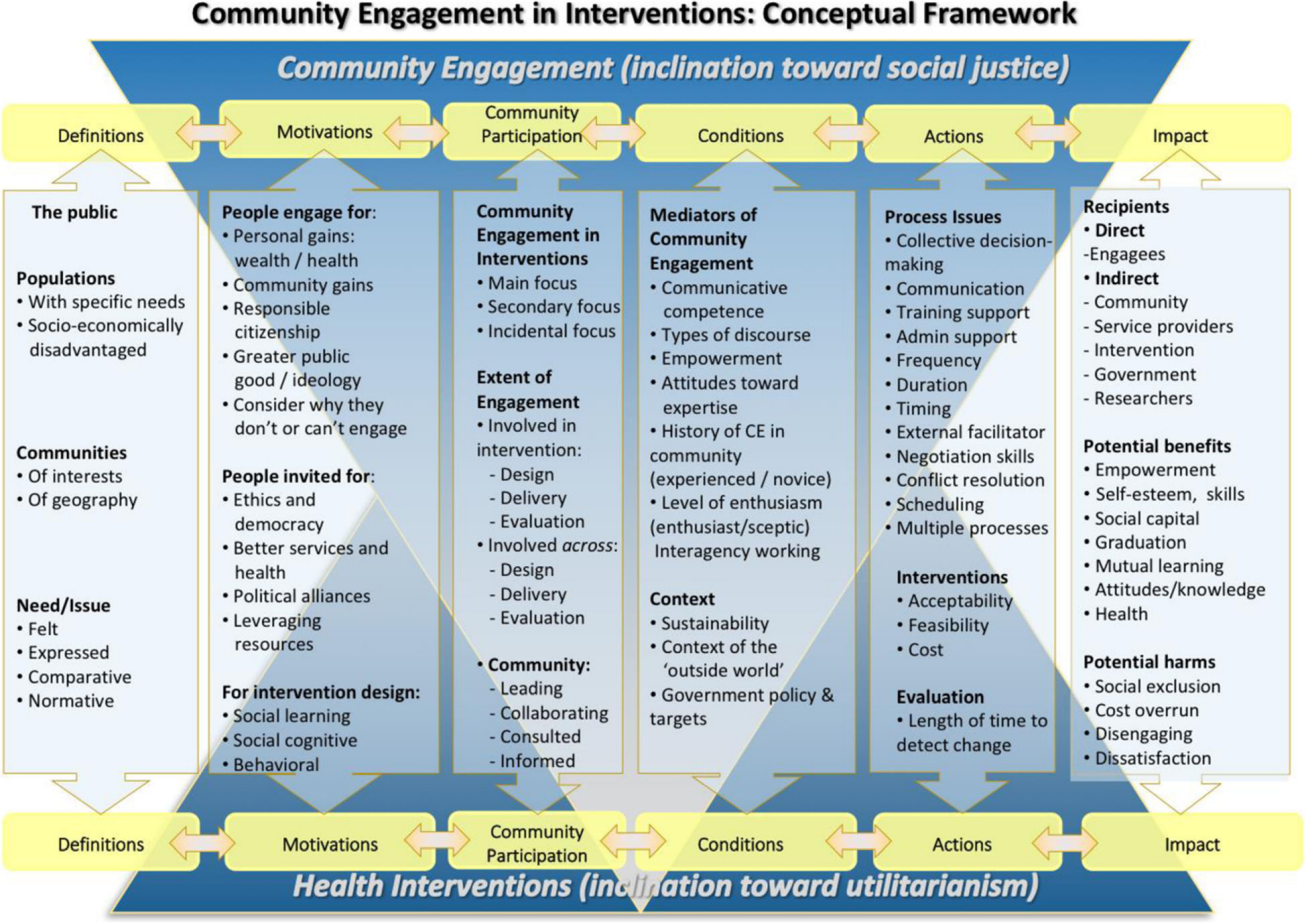
Final conceptual framework

The first task, identifying key concepts and theoretical stances, involved looking at each paper and considering its place in the framework. For example, we needed to understand how ‘community’ was conceptualised in each paper, and their members’ motivations for engagement. The data collected here largely populated the first and second columns of the final framework (Fig. 2). An important aspect of theory synthesis is the ‘translation’ of concepts between studies and settings, which also occurred at this stage. For example, ‘consultative’ activities needed to be labelled consistently across studies; this involved reading studies critically and considering whether a given activity really involved consultation, or was perhaps closer to ‘information provision’ when placed in the context of our emerging framework.

#### Developing models

The second task involved consideration of the relationships between concepts. Here we linked chains of concepts together in order to encapsulate the key arguments made in the literature. For example, we needed to consider how a process of collective decision-making influenced people’s motivations for engagement, and how this in turn might lead to particular outcomes – including harms - for example, disillusionment when expectations were not being met.

The final phase involved both the development of an overarching framework (Fig. 2), and the articulation of specific models which navigated significant paths through the framework. Here, the conceptual framework acted as a system of constructs where some relationships were understood. We pulled out different constructs out of that based on theories (e.g. social justice) to test specific relationships. Authors did not always clearly specify their underlying theory, but their theoretical stance could be inferred based on the context of the study presented. The models were informed by the theory synthesis but operationalized by grouping studies together in different combinations based on their assigned codes for ‘public-identified health need’, ‘involvement in design’ and ‘involvement in delivery’. Multiple combinations were tested before the final operationalization was determined. This process was iterative, involving discussions within the team and our Advisory Group; the development of many ‘trial’ frameworks; and the graphical depiction of the final framework and models. During iteration, different types of intervention were selected purposively to test the framework and to check that its coverage of the approaches present in the included interventions.

### Quality assurance

At each stage of the review (i.e. searching, screening, coding, synthesis), at least two researchers developed, tested and came to agreement on tools and processes using a subset of studies, then independently completed that stage of the review. Queries or disagreements on methods were resolved through discussion with a third member of the review team. Each review stage was conducted using EPPI-Reviewer 4 [13].

## Results

### Included studies and papers

We purposively selected a total of 39 systematic reviews, exemplar process evaluations and theoretical papers that focused on community engagement and provided rich and unique information to develop the conceptual framework. These are listed in Appendix. In addition, a total of 319 included intervention studies of community engagement were also examined for key concepts and patterns of engagement. More details of the flow of studies are described in the full report [5]. Concepts from these reports were extracted into the conceptual framework development and simultaneously considered in the synthesis of theory and development of models. Please see the NIHR report for full details of the results of our searches [5]. From these, three synthesis ‘products’ emerged: (i) theoretical meta-narratives indicating how community engagement is conceptualised across the literature; (ii) theory of change models that operationalised the theoretical meta-narratives; and (iii) an overarching conceptual framework built on the findings from the first two products.

### Significant concepts and definitions within community engagement

As outlined in the methods, the first task in the iterative development of our conceptual framework shown in Fig. 2 involved the identification and definition of significant concepts in the literature.

These were grouped into a set of dimensions which enabled us to explore and categorise differences between the community engagement approaches utilised by the interventions: the extent to which they were concerned with community engagement broadly or health outcomes more narrowly; who it was that identified the need for the intervention; the reasons as to why people might be motivated to become involved; how and where the community was involved in the design and delivery of the intervention; the conditions which mediated or moderated engagement; the types of actions and resources involved in engagement activities; the impacts of the intervention in terms of outcomes and beneficiaries, and their long term sustainability (e.g., programme continuation or the adaptation of programme ideas through other local infrastructure). Each included study addressed one or more of the concepts within each dimension, and across the set of studies we noted that interventions appeared to progress in an iterative fashion through these dimensions from defining the community to considering the impacts. The dimensions are depicted in the vertical columns of the framework shown in Fig. 2.

### Definitions

Community engagement occurs where a need is identified for a particular group of individuals (i.e., a community). Thus the process begins with the definition of both the community and their health issue. Community can be defined in many different ways. In addition to geographical boundaries, they may also be defined by social or economic characteristics, interests, values, or traditions. Such communities (i.e., those with a shared identity, such as the Bangladeshi community, or a shared experience, such as teenage mothers) were the focus of the majority of the included community engagement interventions.

Communities were more likely to define themselves as such, or they might be defined by people outside the community, often labelled as a population. This reflects some semantic differences in how communities were perceived, both by themselves and by external organisations. This distinction between the terms ‘population’ (externally defined) and ‘community’ (self-identified) is shown in the framework.

The health need may also be identified differently [14]:

- a felt need, which is one directly identified by community members themselves;
- an expressed need, which is inferred by observing a community’s use of services;
- a comparative need, derived by comparing service use in a similar community; or
- a normative need; derived by comparing measures of living conditions with a society norm or standard, often set by experts.

This taxonomy delineates different forms of need, which are conceptualised as being on a continuum that moves in stages away from expressly community-identified models (felt need) towards expert opinion (normative need). Across the set of included studies, the community was not involved in establishing need for most interventions: only one quarter of the studies described community involvement in identifying the health need.

### Motivations

Multiple factors can motivate community members to participate in, and professionals to undertake intervention design, delivery or evaluation. These factors depend on the interplay between community engagement and health interventions. Community members might choose to engage for a range of health-related personal, communal and societal reasons, including: personal gains, including monetary/wealth, health and the development of new marketable skills and capabilities; benefits to their community; better community neighbourhoods; less crime; improved educational outcomes; or for the ideals of responsible citizenship, altruism and the greater public good [15–21].

In other cases, those external to the community are motivated to develop a health intervention, driven by their professional responsibilities as, for example, local or state government officials, health care providers, or other community members. Community engagement is fostered here when those within a specific community are invited to participate by those with professional responsibilities. These external stakeholders can ask community members to participate for a broad range of reasons, including: ethics and democracy; the desire to provide better services and better health; for political alliances or to satisfy a political climate; and to leverage resources and increase the chance of sustainability [22–24] (Morison 2000 p.119, in [25]). Involving specific communities as stakeholders can help build public commitment to a health promotion agenda and can empower the public to advocate for change. Such involvement can also help determine whether or in what form a health promoting action is likely to be acceptable for implementation. It may be recognised that some local community groups may be more competent in delivering health promotion change or they may already be involved in other health promoting actions [26]. In other cases, there may be legislative or regulatory requirements for a broader group of individuals to participate; for example, in situations where statutory funding is forthcoming only when matched funding in cash or in-kind is provided by community partners.

It is possible that, even in highly engaged communities, the motivation to continue to participate in developing and implementing an initiative may diminish over time without sufficient financial or other recompense for participation. This may be particularly so for socio-economically deprived or financially constrained communities (e.g., those experiencing low retirement income or requiring paid childcare).

Community engagement initiatives that focus more on health interventions and less on community are often grounded in a specific theory employed by researchers to understand the ways in which people develop, think or act. Examples of theories that motivated intervention design include social learning [27], social cognitive [28, 29], social ecological [30, 31], coalition [32], diffusion of innovation [33], social network [34] or behavioural theory [35]. It is argued throughout the literature that public health interventions should be based on theory that is relevant to, and appropriate for, the population involved, because it can facilitate the examination of constituent intervention components, support the applicability of an intervention with different populations, and ensure a more successful and sustainable intervention through understanding how a community may be moblised [36–43].

### Community participation

The definitions, needs and motivations of communities provide a foundation to structure how community engagement is developed and delivered. Where community engagement is a key part of the strategy, members of the stakeholder community can be involved in the design of an intervention [42, 44]. Conversely, where there is less community engagement and more emphasis on a health intervention, members may simply take part in its delivery [45]. The number of people taking part in the community initiative can influence the level of engagement that takes place [46]. These levels of engagement can be thought of as hierarchical, progressing from least to most engagement: receiving information; consultation; collaboration; and control [47].

Studies in this synthesis also varied considerably with respect to the extent to which community engagement is ‘embedded’ as a predetermined, planned part of a health intervention. It may vary from being the main focus of the intervention, as in local area regeneration programmes [15], to operating as an important secondary part of the intervention in which the main intervention is supported by, but not dependent on, community engagement. An example of this is a community-informed food labelling system offered within a complex community cardiovascular disease prevention strategy [42]. In other cases, those currently in positions of power may need to be ‘engaged’ in interventions in order to empower a disadvantaged community, thus enabling it to improve its own health [19, 48]. The community engagement mechanism may also occur through intervention delivery, such as in the use of peers or lay health advisors to deliver health messages [45].

### Conditions

Several included studies discussed the contextual influences or mediators necessary for community engagement initiatives. These included communicative competence [22–24]; empowerment and control [49–51]; and attitudes by community members and providers towards what expertise was important and who held it [15, 52]. The extent to which communities can engage appeared to be dependent on the level of financial and other resources available to support their participation [53, 54].

The context in which a community engagement initiative or health intervention took place also influenced its impact on health. Contextual issues included the degree of stable funding and support throughout the project [15, 55] and the level of certainty over future funding or mainstreaming opportunities [20]; the social, political, economic, geographic context and its impact on the community engagement or public health interventions [16, 56, 57]; and the influence of externally-imposed government policy and targets for achieving health [58]. The extent to which a community engagement initiative has to compete for resources and visibility with other national/local health promotion initiatives was also identified as an important contextual factor [21]. In addition, changes in the local economic climate may influence communities’ ability and/or interest in participating. The nature and impact of these influences may only be captured if a process evaluation is conducted.

Many of these conditions are thought to create (or fail to create) an environment for the development of virtuous (or vicious) circles. In this environment, some of the facilitators described above mutually reinforce one another and help the initiative to become self-sustaining. In situations where trust is lacking, or no previous history of collaboration exists, engagement can be difficult to achieve and will have little momentum in terms of sustainability [19]. These feedback loops are often seen in complex interventions and may bring disproportionate rewards. For example, at particular critical levels ‘tipping points’ may be reached, whereby a small increase (or decrease) in resource can bring about a disproportionate change in outcomes [59].

### Actions

The way in which a community engagement activity takes place (i.e., the ‘process’ of engagement) is thought to influence how well that activity ultimately impacts on health outcomes. Several examples of process issues were discussed in the literature. These included:

- clearly defined target groups, objectives, interventions and programme components [46, 60];
- adequate time for community members and other stakeholders to build relationships with one another, so that they can agree a ‘level playing field’ in terms of language, negotiation and collegial working skills [17, 24, 25, 61];
- learning of funding sources and developing skills to bid for future sources of funding [21];
- the degree of collective decision-making [15, 16, 52];
- planning for on-going simple communication between participants and providers [39, 49–51], and between the community engagement group and the wider community [36, 49–51, 57];
- adequate participant and provider skills training [16, 17, 25, 36, 45, 46, 49];
- the amount and quality of administrative support required to ensure smooth project running [49, 57, 62];
- activity timing, duration and frequency [39, 58, 61, 63]; and
- cash flow stability throughout the lifetime of the initiative [64].

### Impacts

While the included literature suggests that understanding and planning for key stages in the process of community engagement may impact on outcomes, it also suggests that who is affected, and in what ways, should be considered. For example, South and colleagues [65] suggest that a range of people can benefit from community engagement and/or public health interventions. These can be described as ‘direct’ or ‘indirect’ beneficiaries. Direct beneficiaries are those who take part in the community engagement (the ‘engagees’). In this case, the act of being engaged is the intervention for which outcomes are measured. These can be health outcomes, empowerment, self-esteem, skills development, level of interest, learning activities and gains [57, 60, 62].

In contrast, indirect beneficiaries are the wider community toward whom community engagement and/or public health interventions are targeted, or the service providers who engage with the communities [66]. Both of these indirect beneficiaries benefit by mutual learning. Researchers can also be considered indirect beneficiaries, in that further research and interventions can be perpetuated from a community engagement initiative. Government departments might benefit by being able to demonstrate that their policies made a difference (i.e., targets were met), or that a particular political priority was successful [66]. The intervention itself can benefit from the amount and type of community engagement: interventions can be sustained and improve with community engagement [66]. The type of outcomes measured on indirect beneficiaries can include health outcomes and social capital. Evaluated community engagement interventions may be cost effective, taking into account impacts on engagees and the community of interest. This is particularly the case when multiple health and non-health benefits of engagement are taken into account [20, 67].

Some harms potentially resulting from community engagement were identified, especially when communities are less involved. These included social exclusion, cost overrun, attrition, and dissatisfaction and disillusionment [56, 64, 66]. It has also been suggested that community partners and decision-making organisations should collaborate to strike a balance between ‘soft’ relational outcomes and ‘hard’ policy impacts [56].

In determining these concepts as described by authors across the retrieved studies, we noted that some of them appeared to arise from a desire to engage communities, whilst others appeared to be driven by a desire to intervene in order to improve a community or populations’ health. These two areas are represented by the inverted triangles in Fig. 2 labelled as ‘Community engagement’ and ‘Health intervention’.

### The two schools of thought within “community engagement”

Community engagement has been advanced as actions ‘involving communities in decision-making and in the planning, design, governance and delivery of services’ [68], and is a potentially promising strategy to promote health and healthcare [1]. Several strategies have been suggested to engage different communities to varying degrees. Some have suggested that involvement comprises consultation, collaboration, or community control, with the provision of information alone not considered a sufficient level of engagement [47]; others have suggested that community engagement taxonomies should also include information-giving [69]. Community engagement can occur alone or in combination with other initiatives; however in the latter case, its unique contribution to changes in outcomes may be difficult to establish [70]. Community engagement activities are consequently diverse, and in the UK include but are not limited to: service user networks; healthcare forums; volunteering; and courses delivered by trained peers [71].

Two clear perspectives, or ‘meta narratives’ emerged which explained why community engagement might improve people’s health: a health services, or ‘**utilitarian**’ perspective; and a ‘**social justice**’ perspective. Historically, interventions to promote health were driven by professionals, with little or no input from the targeted populations [72]; more recently, community engagement has become central to national strategy and guidance for promoting public health, because, from a ‘utilitarian’ point of view, it is thought that more acceptable and appropriate interventions will result, which may result in improved service use and outcomes [2].

As well as the ‘discovery’ of community engagement by the health services and policy community, the literature also describes a distinct tradition of community engagement which is rooted in ‘social justice’ and civil rights. Here the emphasis is less on an instrumental use of community engagement to achieve a given end, but on the empowerment and development of the community itself. These two perspectives, and approaches that bridge the two perspectives, are detailed below.

#### A utilitarian health systems perspective

Interventions that are based on a utilitarian perspective seek to involve communities in order to improve the effectiveness of the intervention. The intervention itself may be decided upon before the community is invited for its views; or, while the intervention itself is not designed by community members they may be involved in other ways, such as priority setting, or in its delivery. In utilitarian perspectives, health (and other) services reach out to engage particular communities that they have identified require assistance and the intervention is devised within existing policy, practice, and resource frameworks.

The large number of studies we found in which peers or lay people delivered the intervention exemplify utilitarian interventions. The content of these interventions did not usually change in their delivery; however, it was thought that peers could deliver that content in such a way that it would be more effective due to their credibility, empathy, community contextual awareness, and so on.

#### A social justice perspective

‘Empowerment’ is rooted in concerns about social justice and movements promoting social and structural change by supporting people to participate, negotiate, influence control and hold accountable institutions that affect them. It is considered socially desirable, equitable and addresses some of the social determinants of ill health, and thus will also result in improved health and reductions in health inequalities. Empowerment models require that the health need is identified by the community and that they mobilise themselves into action. An empowered community is the product of enhancing their mutual support and their collective action to mobilise resources of their own and from elsewhere to make changes within the community. From a social justice perspective, community members are empowered to determine for themselves the priorities and ways in which they want service resources to be deployed. While the ultimate aim may be improvements in health, the social justice agenda is broader than this, and concerned with making up deficits in power, democracy and accountability.

In this literature, terms such as ‘engagement’, ‘participation and ‘development’ can sometimes be used interchangeably, with the World Health Organisation defining community ‘development’ as: “A way of working underpinned by a commitment to equity, social justice and participation that enables people to strengthen networks and to identify common concerns and supports people in taking action related to the networks. It respects community-defined priorities, recognizes community assets as well as problems, gives priority to capacity-building and is a key mechanism for enabling effective community participation and empowerment.” [73].

‘Arnstein’s ladder’ is one of the best known models based on social justice, showing how different models of participation are more or less empowering than others (Arnstein 1969). It begins with essentially ‘non-participative’ ways in which those holding power can reach out to those who do not, and ends with ‘citizen control’, in which power to direct has been ceded or been devolved completely. In this model true participation only begins once power is delegated or developed, with other types of participation being dismissed as ‘tokenism’ and ‘non-participation’. It is important to recognise the ethical and political dimension of the ladder. As well as representing ‘effective’ ways to involve the public in public policy (and to improve the nations’ health), the top of the ladder represents more democratic and egalitarian approaches towards public service, whereas the lower rungs tend to be associated with authoritarianism and a lack of accountability.

#### Bridging the utilitarian and social justice rationales

These two perspectives often collide in the literature on community engagement, as authors take differing positions, depending on the tradition within which they are writing. The fact that there are two traditions of thought and objective in this literature means that the term ‘community engagement’ can be used differently by different authors, depending on their conceptual location, leading one researcher to conclude:

‘…the proliferation of meanings attached to the phrase “community participation in health”… has allowed it to be analysed as a political symbol capable of being simultaneously employed by a variety of actors to advance conflicting goals, precisely because it means different things to different people .’[73]

Many models, however, merge the above two perspectives, arguing for community engagement for utilitarian purposes as well as for social justice. Indeed, they reason that, since the relatively poorer health of disadvantaged groups is due to structural issues – over which they have limited control – an effective way of improving their health will be to cede power to these communities in a way that helps them to change their environment for the better. However the concepts of utilitarianism or social justice were rarely directly addressed by authors. An example of this can be demonstrated by Barnes et al. in which community volunteers provided an outreach, tracking and follow-up program in response to high under-immunisation rates amongst an urban New York population [74]. Here, community members were ‘committed and organised’; they identified the need for the program, led on the design and delivery of the intervention and collaborated on its evaluation, suggesting that these community members were empowered in doing so.

Popay et al. [75] argue that the ‘pathways from community engagement to health improvement’ is a good example of this model. In it, they argue, significant changes to people’s health outcomes require changes to ‘intermediate social outcomes’: improved social capital and social and material conditions. However, changes to these intermediate outcomes are only triggered once sufficient power has been ceded: information and consultation are not sufficient; only once a level of co-production has been reached do these begin to move, and it requires delegated power and full community control for the highest gains to be realised.

### Models in community engagement

The theory synthesis building on the initial conceptual framework identified a wide range of dimensions by which community engagement interventions may differ from one another, and provides a structure to understand how different interventions may function and different components combine and interact as a whole. While there are many ways in which the different dimensions might be arranged, our theoretical synthesis suggested that those falling into the social justice and utilitarian theoretical meta-narratives were found to be important in the interventions identified in the review; and intermingling of these two were found throughout the literature. From this conceptual framework, we identified clusters of concepts that prompted us to develop three hypothesised models:

#### ‘Classical’ or ‘traditional’ peer- or lay-delivered interventions

In these interventions, specific health needs and relevant populations are identified usually by normative or comparative methods, and peers or lay people recruited in order for the intervention to be delivered in the most appropriate way for the population. The delivery of the intervention is thus thought to be more empathetic and credible (and resulting outcomes better) because of delivery by members of the community. Communities do not participate in the design of the intervention, and the theory of change focuses on communicative and implementation competence rather than empowerment or people’s attitudes towards expertise. Beneficiaries are usually understood at the individual, rather than community, level, and the people delivering the intervention themselves have often been found to benefit significantly. Sometimes these interventions have been reported to be cost-effective compared to no-action and/or professionally delivered services [76–78].

#### Interventions with varying degrees of collaboration between health/other statutory services and communities

**Fig. 3:**
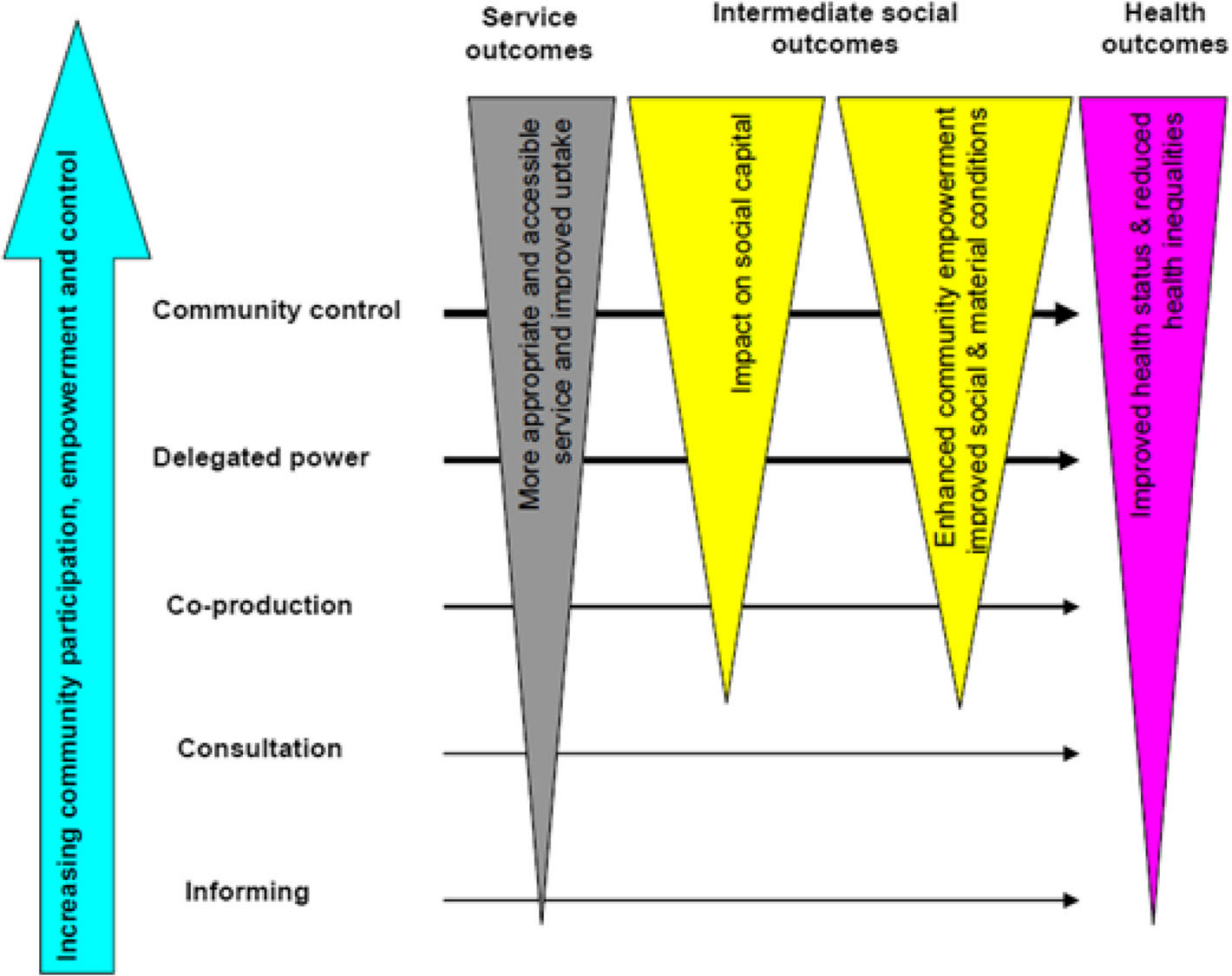
Varying degrees of collaboration between health/other statutory services and communities From Popay et al. (2006) [76]

As discussed above, a wide range of models are concerned with engaging the community in intervention design and implementation. This involvement can range in the extent of community participation, empowerment and control, influencing service, intermediate social outcomes and health outcomes, illustrated in Fig. 3 [72].

Need is usually identified by people outside the community (‘expressed’, ‘comparative’ or ‘normative’), but the theory of change includes specific community engagement in order to better align the intervention to the community’s needs and preferences. The extent of community involvement in the intervention can vary considerably: the framework describes a range of dimensions reflecting this variability (e.g., whether the community leads on designing or delivering the intervention, and who the beneficiaries are). The theory of change developed by Popay et al. [76] depicted in Fig. 3 reflects this model and suggests that ‘degree of engagement’ may be a useful analytical approach. “The diagram highlights four broad approaches to community engagement differentiated by their engagement goal: the provision and/or exchange of information; consultation; co-production; and community control. These approaches are not readily bounded but rather sit on a continuum of engagement approaches with the focus on community empowerment becoming more explicit and having greater priority to the right of the continuum where community development approaches are located.” [75].

#### Interventions based on empowerment

Sometimes a subset of the second model above, this set of interventions is distinguished from others because the need for these interventions was identified by the community itself [79, 80]. The community will often have a collaborating role in designing the intervention and the underpinning theory of change is around empowering communities to make changes to their social and environmental locales [81]. These initiatives may not be focused exclusively on improving people’s health, as they may be addressing more issues – of which health is but one outcome. In terms of its contribution to our framework, empowerment is understood both as an outcome and as a ‘mediator’, as empowerment is thought to improve a range of interventions (as per the second model above) as well as being a specific aim of others.

## Discussion

The synthesis presented in this report is part of a larger systematic review, which comprehensively examined the models, practice, outcomes and economics of using community engagement to improve the health of disadvantaged groups. A major contribution of this work is its ability to compare different ways of providing community engagement and some potential underlying models. A variety of intervention strategies were identified which we suggest could be broadly understood as drawing on different combinations of both utilitarian (health systems) and social justice (ideological) perspectives. We have found no other systematic reviews that have synthesised evidence representing such a broad spectrum of community engagement models that span the utilitarian-social justice divide.

Our work has produced [1] a conceptual framework that illustrates the wide range of concepts thought to influence community engagement, [2] a range of resultant models expressing different concepts from the framework, and [3] the suggested underlying perspectives that drive those models. The meta-analysis examining the effectiveness of community engagement suggests that interventions developed from both utilitarian and social justice perspectives tend to demonstrate effectiveness [3]. Importantly, this also allows us to consider which community engagement approaches might be more effective under different circumstances, rather than constraining our thinking to models that conform to specific underlying theories.

That is, the conceptual framework and the models encourage a fit-for-purpose approach to designing community engagement interventions because they embrace diversity and promote thinking about dimensions of difference across health definitions, motivations, participation models, conditions, actions, and impacts [3, 82].

As many authors have observed, ‘community engagement’ suffers from a bewilderingly large number of inconsistent and partially conflicting definitions [75, 83, 84]. We have not re-defined these, nor added a new one to the already extensive catalogue; rather, we have sought to understand the perspectives behind some of the more significant definitions, what they mean in practice, and to characterise them in terms of their different models. We hope this will complement existing definitions and aid future evaluations and evidence syntheses by suggesting that, rather than focusing on the overarching heterogeneous concept of community engagement, we may be better served by identifying the key characteristics of interventions and how these relate to their underpinning models. Indeed, they have already been used in examining the effectiveness and cost-effectiveness of diverse types of community engagement in public health activities [5, 85]. Moreover, whilst this was framework was developed in the context of public health, it has conceivable applicability to other areas including education and schools, policing and criminology, public transport services, the environment, and other areas in which the community could make a meaningful contribution or have a stake in the service provided.

## Conclusions

We sought to capture all the concepts that were discussed by authors as important to community engagement interventions, then considered iteratively the theoretical underpinnings of the interventions that utilised community engagement in order to identify the models common to most of the interventions included in the review. This is meant to help researchers, community members and public health professionals to understand their own (often unexamined) philosophy underpinning the interventions they are considering. It also helps them to choose from a wider group of conceptual options than they might otherwise know about. This also provides those evaluating community engagement initiatives with a wider range of criteria (for example, were community members informed, consulted, or did they collaborate or lead?).

The theoretical synthesis, conceptual framework and the models presented here are useful tools for researchers, community members and public health professionals who are considering appropriate and effective approaches to community engagement. The theoretical synthesis makes clear the two schools of thought driving community engagement, the overlap of these philosophies in the operationalization of the resulting interventions, and the utility of considering the theory of change to understand these different starting points of the interventions.

Our new heuristic for understanding the dimensions of community engagement (i.e. the conceptual framework) should assist those developing interventions in the future to align their strategy with an appropriate theory of change. These conceptual tools should be considered, discussed, tested and adapted by researchers in order to facilitate intervention design and evaluation, and further theory testing.

Public health professionals could use the conceptual framework to capture specific aspects of the economic- and process-related aspects of community engagement. This will help to disentangle the relative effectiveness of different models of community engagement and so promote effective, sustainable and appropriate community initiatives.

## Appendix

### References informing the theory synthesis, conceptual framework and models (*N* = 39)

Anastacio J, Gidley B, Hart L, Keith M, Mayo M, and Kowarzik U. (2000). *Reflecting realities: Participants’ perspectives on integrated communities and sustainable development*. Bristol: Policy Press, pp.1–59.

Arblaster L, Entwistle V, Lambert M, Forster M, Sheldon T, and Watt I. (1995). *Review of the research on the effectiveness of health service interventions to reduce variations in health.* York: NHS Centre for Reviews and Dissemination, pp.157.

Attree P. (2004). ‘It was like my little acorn, and it’s going to grow into a big tree’: a qualitative study of a community support project. *Health Soc Care Community*, 12(2), pp.155–61.

Barnes M, Newman J, Knops A, and Sullivan H. (2003). Constituting ‘the public’ in public participation. *Public Administration*, 81(2), pp.379–399.

Barnes M, Sullivan H, and Matka E. (2004). *The development of collaborative capacity in Health Action Zones: A final report from the national evaluation*. Birmingham: University of Birminham.

Barnes Marian, Knops Andrew, Newman Janet, and Sullivan Helen. (2004). Public participation and collaborative governance. *Journal of Social Policy*, *33(2)*, pp.203–223.

Beresford P, and Hoban M. (2005). *Effective participation in anti-poverty and regeneration work and research*. York: The Joseph Rowntree Foundation and Brunel University. Available at: https://www.jrf.org.uk/report/effective-participation-anti-poverty-and-regeneration-work-and-research.

Bickerstaff K, and Walker G. (2005). Shared visions, unholy alliances: Power, governance and deliberative processes in local transport planning. *Urban Studies*, 42, pp.2123–2144.

Birchall J, and Simmons R. (2004). *User power: the participation of users in public services*. London: National Consumer Council. Available at: http://hdl.handle.net/1893/3261.

Blankenship K M, Bray S J, and Merson M H. (2000). Structural interventions in public health. *AIDS*, 14, pp. S11-S21.

Campbell R, Starkey F, Holliday J, Audrey S, Bloor M, Parry-Langdon N, Hughes R, and Moore L. (2008). An informal school-based peer-led intervention for smoking prevention in adolescence (ASSIST): a cluster randomised trial. Lancet, 371(9624), pp.1595–602.

Chouhan K, and Lusane C. (2004). *Black voluntary and community sector funding: Its impact on civic engagement and capacity building.*. York: Joseph Rowntree Foundation.

Church C, and Elster J. (2002). *Thinking locally, acting nationally: lessons for national policy from work on local sustainability*. London: Joseph Rowntree Foundation. Available at: https://www.jrf.org.uk/report/lessons-local-action-national-policy-sustainable-development.

Evans D, Pilkington P, and McEachran M. (2010). Rhetoric or reality? A systematic review of the impact of participatory approaches by UK public health units on health and social outcomes. *Journal of Public Health*, 32(3), pp.418–426.

Fisher E B.,Jr., Auslander W, Sussman L, Owens N, and Jackson-Thompson J. (1992). Community organization and health promotion in minority neighborhoods. *Ethn.Dis.*, 2(3), pp.252–272.

Flowers P, Hart GJ, Williamson LM, Frankis JS, and Der GJ. (2002). Does bar-based, peer-led sexual health promotion have a community-level effect amongst gay men in Scotland? *International Journal of STD & AIDS*, 13(2), pp.102–8.

Goodlad R, Burton P, and Croft J. (2005). Effectiveness at what? The processes and impact of community involvement in area-based initiatives. *Environment and Planning C-Government and Policy*, 23, pp.923–938.

Hamer M, and Box V. (2000). An evaluation of the development and functioning of the Boscombe Network for Change, a health alliance or partnership for health in Dorset. *Health Education Journal*, 59, pp.238–52.

Holder H D, and Moore R S. (2000). Institutionalization of community action projects to reduce alcohol-use related problems: systematic facilitators. *Subst Use Misuse*, 35(1–2), pp.75–86.

Jemmott L S, Brown E J, and Dodds S. (1998). Building community partnerships to improve HIV prevention efforts: implications for nurses. *J Assoc Nurses AIDS Care*, 9(3), pp.29–40.

Ltd. EDuce. (2005). *Seeking the Lessons: Skills and Knowledge Programme Evaluation.*. London: EDuce, Office of the Deputy Prime Minister. Available at: http://www.eukn.eu/e-library/project/bericht/eventDetail/seeking-the-lessons-skills-and-knowledge-programme-evaluation-uk/.

Matthews H. (2001). *Citizenship, youth councils and young people’s participation.* London: Joseph Rowntree Foundation/Policy Press.

McInroy N, and MacDonald S. (2005). From community garden to Westminster: Active citizenship and the role of public space. Manchester: Centre for Local Economic Strategies.

Office of the Deputy Prime Minister & Neighbourhood Renewal. (2005). *Views of New Deal for Communities – focus group report*. London: Office of the Deputy Prime Minister.

Osborne S, Beattie R, and Williamson A. (2002). *Community involvement in rural regeneration partnerships in the UK: Evidence from England, Northern Ireland and Scotland*. Bristol: The Policy Press and the Joseph Rowntree Foundation. Available at: https://www.jrf.org.uk/report/community-involvement-rural-regeneration-partnerships-uk-evidence-england-northern-ireland.

Popay J, Attree P, Hornby D, Milton B, Whitehead M, French B, Kowarzik U, Simpson N, and Povall S. (2007). *Community engagement in initiatives addressing the wider social determinants of health. A rapid review of evidence on impact, experience and process*. Lancaster: Universities of Lancaster, Liverpool and Central Lancashire, pp.1–222.

Ritchie D. (2001). ‘Breathing Space’: Reflecting upon the realities of community partnerships and workers’ beliefs about promoting health. Health Education Journal, 60(1), pp.73–92.

Ritchie D, Parry O, Gnich W, et al. (2004). Issues of participation, ownership and empowerment in a community development programme: tackling smoking in a low-income area in Scotland. Health Promot International, 19(1), pp.51–9.

Robinson N, and Lorenc AR. (2010). *Strengthening the public voice in shaping sexual and reproductive health services - Changing relationships*. London: London Sexual Health Programme, pp.115.

Russell H. (2005). *Voluntary and Community Sector Engagement in Local Strategic Partnerships: National Evaluation of Local Strategic Partnerships Issues Paper*. London: Department of Transport and Office of the Deputy Prime Minister.

Seyfang G, and Smith K. (2002). *The time of our lives: Using time banking for neighbourhood renewal and community capacity building*. London: New Economics Foundation. Available at: https://ueaeprints.uea.ac.uk/29416/.

Shiner M, Thom B, MacGregor S, Gordon D, and Bayley M. (2004). *Exploring community responses to drugs*. York: Joseph Rowntree Foundation and London School of Economics and Political Science. Available at: https://www.jrf.org.uk/report/exploring-community-responses-drugs.

Sridharan S, Gnich W, Moffat V, Bolton J, Harkins C, Hume M, Nakaima A, MacDougall I, and Docherty P. (2008). *Independent Evaluation of Have a Heart Paisley Phase 2*. Edinburgh: University of Edinburgh.

Swainston K, and Summerbell C. (2008). *The effectiveness of community engagement approaches and methods for health promotion interventions*. University of Teeside, pp.1–226.

Taylor M, Ardon R, Carlton N, Meegan R, Purdue D, Russell H, Syed A, and Wilson M. (2005). *Making Connections: An Evaluation of the Community Participation Programmes*. London: Office of the Deputy Prime Minister. Available at: http://eprints.uwe.ac.uk/10168/.

Taylor Marilyn. (2006). Communities in Partnership: Developing a Strategic Voice. *Social Policy and Society*, 5(02), pp.269–279.

Treno AJ, and Holder HD. (1997). Community mobilization: evaluation of an environmental approach to local action. *Addiction (Abingdon, and England)*, 92 Suppl 2, pp.S173–87.

Weinehall L, Hellsten G, Boman K, and Hallmans G. (2001). Prevention of cardiovascular disease in Sweden: the Norsjo community intervention programme--motives, methods and intervention components. *Scand J Public Health Suppl.*, 56:13–20., pp.13–20.

Williams Mike. (2004). Discursive Democracy and New Labour: Five Ways in Which Decision-Makers Manage Citizen Agendas in Public Participation Initiatives. *Sociological Research Online*, 9(3). Available at: http://www.socresonline.org.uk/9/3/williams.html
